# Anthropogenic impact assessment on the water quality of Umhlathuze River in KwaZulu Natal Province, South Africa

**DOI:** 10.1007/s10653-025-02830-0

**Published:** 2025-10-21

**Authors:** Makhosonke Simon Bhengu, Pinkie Ntola, Somandla Ncube

**Affiliations:** Department of Chemistry, Durban University of Technology, P O Box 1334, Durban, 4000 South Africa

**Keywords:** Water quality, Metal pollution, Umhlathuze river, Health risk assessment, Hazard quotient

## Abstract

Umhlathuze River and its three main tributaries experiences various anthropogenic activities including agriculture, urbanization and industrialization before forming an estuary with the Indian ocean. The current study assessed the potential impact of these anthropogenic activities over a 12–months period in 2023/4. The study found that elementary levels were mostly within the permissible limits along Umhlathuze River, but the impact of anthropogenic activities along its tributaries was evident. Chlorides ranged between 400 and 655 mg L^−1^ along the Mholweni tributary during the rainy season while Na, Mg and Ca were also consistently above permissible limits along Mhloweni and Ntambanana tributaries. Among non–toxic trace elements, only Fe exceeded its health–based precautional value during the rainy season for all sites. Trace toxic metal levels were also within permissible limits. Hg was only detected along the tributaries in the 0.017–0.399 µg L^−1^ range. The source of elevated levels of parameters along the tributaries was linked to quarry and agricultural activities, the wastewater effluents and urban runoff. The WQI values were in the 10.7–36.1 range implying the river water could be classified as good to excellent. The main river water was also found to be suitable for irrigation purposes, but water from the three tributaries was unsuitable with SAR values of 29.5–76.3. A health risk assessment identified that the river water was safe for adults but infants and children were more likely to develop non–carcinogenic effects with HI values of 1.07–3.28. The ILCR values ranged between 1.20 and 9.93 indicating that consumption of unprocessed water from Umhlathuze and its tributaries will result in carcinogenic effects over one’s lifetime with infants and children affected most.

**Supplementary Information:**

The online version contains supplementary material available at 10.1007/s10653-025-02830-0.

## Introduction

Anthropogenic processes have been reported to contribute to metal pollution of river water systems. Developing countries including South Africa still experience a challenge with metal contamination of surface water resources with researchers identifying wastewater effluents and mine runoff as the major contributors of metal pollution in surface water systems (Dzhangi & Atangana, 2024; Harding et al., 2020; Ntombela et al., 2016; van Rensburg et al., 2016). Metals have known health side effects if present above certain levels in drinking water sources (Jomova et al., 2024; Zaynab et al., 2022). Recent studies have been done on metal pollution of important rivers within South Africa with some results reporting metal levels higher than the WHO and the South African Water Quality (SAWQ) guidelines (Edokpayi et al., 2016; Moloi et al., 2020). These observations may still be ignored at legislation level but the potential impact on the aquatic organisms and rural communities that may consume the river water in its raw form cannot be ignored, with more research required to provide more evidence for remedial action.

Umhlathuze River in northern part of KwaZulu–Natal province is currently exposed to anthropogenic activities including the rapidly growing urbanization as well as growth in agricultural and industrial activities all with the potential to release metal elements into the river if not properly managed (Mokoma & Tilahun, 2022). The river has also been impacted by inclement environmental conditions including the drought between 2014 and 2020, extreme floods in 2022 as well as the growth of new chemical industries and urban settlements within its catchment (Dlamini et al., 2024; Mashao et al., 2023). A previous study conducted by Mthembu and colleagues along uMhlathuze River in 2012 investigated six metals (Mn, Pb, Al, Hg, Cu and Cd) at four sampling sites and observed that all the metal contamination levels were higher than the South African Water Quality guideline limits (Mthembu et al., 2012). It is therefore important to perform recent pollution studies to better understand the current state of water quality along Umhlathuze River and assess the impact that these anthropogenic activities and adverse weather conditions could have on the river’s ecosystem.

The current study aimed to provide a recent state of water quality along uMhlathuze River by investigating 22 selected metals at thirteen sites strategically selected along the river and its catchment taking into consideration various anthropogenic activities along the river. The metals included the four major macro elements (Na, Mg, K and Ca); six essential trace metals (Cu, Co, Fe, Mn, Ni and Zn); six toxic trace metals (Cr, Cd, Hg, As, Pb, U). Six other elements (Al, Se, Sb, Ba, B, Sr) with limited effect on the human body were included in the study. In addition, some physicochemical parameters including pH, temperature, and dissolved oxygen were conducted and results were linked to the measured levels of the metals. Other nutrient chemicals like chloride, fluoride, nitrate and sulfate were also included in the study. Principal component analysis was used to understand the interrelationships between the various water parameters and the elementary composition. The measured concentrations were used to predict water quality of Umhlathuze River based on water quality indices (WQI) and heavy metal pollution index (HPI), the suitability of the water for irrigation purposes based on the sodium adsorption ratio (SAR), and health risk using the hazard index (HI) and incremental lifetime cancer risk (ILCR).

## Materials and methods

### Chemicals and reagents

Individual 1000 mg L^−1^ stock solutions (K, Na, Mg & Ca) were purchased from Merck Pty Ltd, Johannesburg, South Africa. A mixed stock solution of Al, As, Hg, B, Ba, Cd, Co, Co, Cr, Cu, Fe, Mn, Ni, Pb, Se, Sr, U and Zn with concentration range of 1000 mg/L was purchased from Separations, Johannesburg, South Africa. The anion standards were also purchased as 1000 mg/L standards from Merck Pty Ltd, Johannesburg, South Africa.

### Instrumentation

A Dionex ICS 1600 Ion Chromatograph by Anatec Suppliers, Johannesburg, South Africa was used for analysis of chlorides, fluorides, nitrates and sulfate. Total organic carbon (TOC) was analysed using Analyticjena Multi N/C 3100 TOC instrument from Spectrometer Technologies, Johannesburg, South Africa. An Inductively Coupled Plasma Optical Emission Spectrometry (ICP–OES) instrument from Perkin Elmer, Johannesburg, South Africa was used for analysis of macro elements. The ICP–OES instrument conditions were RF power 1.5 KW, plasma flow 8 L min^−1^, auxiliary flow 0.2 L min^−1^, nebuliser flow 0.7 L min^−1^, pump rate 1 mL min^−1^. Elemental wavelengths selected were Ca (317.933 nm), Mg (285.213 nm), Na (589.592 nm) and K (766.490 nm). An Inductively Coupled Plasma Mass Spectrometry (ICP–MS) from Perkin Elmer, Johannesburg, South Africa was used to analyse trace metals that included Al, Cu, Co, Fe, Mn, Cr, Cd, Hg, As, Se, Sb, Pb, Ni, Ba, B, U, Sr, and Zn. Instrument conditions for ICP–MS were RF power 1.55 kW, carrier gas 2 L/min, helium flow 4.5 mL min^−1^ and pump rate of –3 rpm. Gas used for both ICP–OES and ICP–MS was argon of 99.999% purity from Air Product, Richards Bay, South Africa.

The calibration range for Ca and Mg was 5–60 µg mL^−1^, for K was 0.25–30 µg mL^−1^ while for Na was 25–200 µg mL^−1^. The calibration range for trace metals on the ICP–MS instrument was 10–1000 ng mL^−1^ except for Hg which was 0.1–50 ng mL^−1^. Both instruments were set to accept the linearity of the calibration curves only if R^2^ > 0.999. In this regard, the R^2^ values for all the elements varied between 0.9995 (Ba) and 1 (Hg). The limits of detection (LODs) and limits of quantitation (LOQs) for the elements were estimated based on the linear regression method using the LINEST function on Excel. The LODs for trace elements ranged between 0.11 and 0.59 ng/mL, while macro elements were in the 0.41–5.46 mg/L range. The LOQs were 0.36 – 1.90 ng/L for trace elements and 0.36–18.2 mg/L for macro elements. The LODs and LOQs for each element are given in the Supplementary. For precision, all calibration standards and samples were run in triplicate, and the average used in further data processing. A relative standard deviation less than 20% (RSD < 20%) was considered acceptable.

### Sampling area

**Fig. 1:**
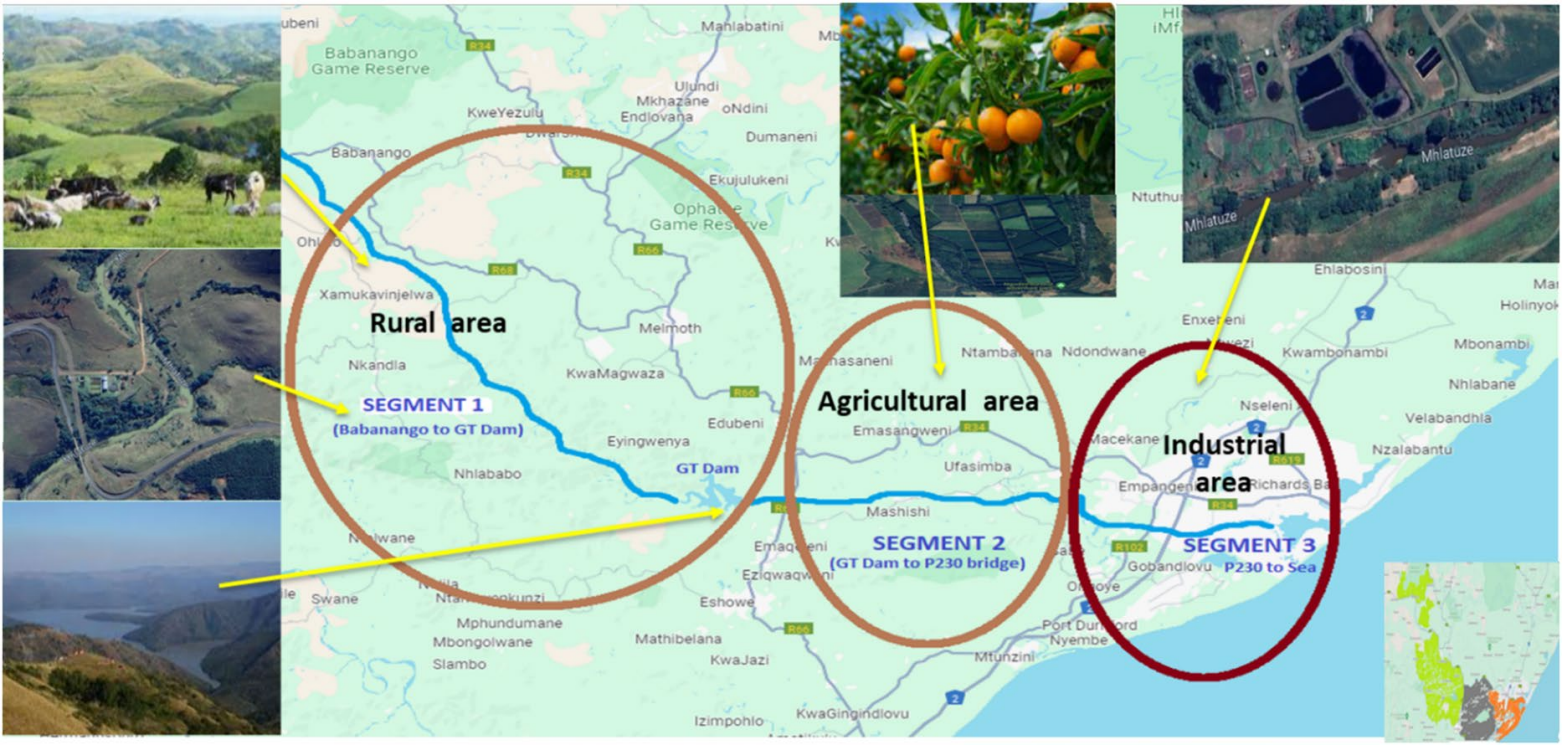
Anthropogenic activities per segment along uMhlathuze River

The uMhlathuze River was divided into three segments based on the anthropogenic activities (Fig. 1). The first segment represented rural areas between Babanango mountains where uMhlathuze River originates, up until Gouderetrouw Dam which is about 60 km before uMhlathuze River meets the Indian Ocean. The second segment is an agricultural region in which Umhlathuze River is surrounded by sugarcane fields and citrus farms and processing factories. In this region Umhlathuze river meets with various tributaries with the two major ones (Mholweni and Ntambanana Rivers) selected for sampling just before they meet UMhlathuze River. Mholweni River emerges from Dlangubo rural areas and passes through a quarry. Ntambanana River originates from Ntambanana rural areas and passes through sugarcane fields. The third segment covers areas around Empangeni Town and Richards Bay until the estuary. This is an urban area with a variety of chemical and processing industries, three wastewater treatment plants and various informal settlements. Thirteen sampling points were strategically selected for this study of which three were in Segment 1, six in Segment 2 and four in Segment 3. The GPS coordinates for each sampling point are given in the Supplementary.

### Sample collection and analysis

Water samples were collected into 250 mL clean polyethylene plastic sampling bottles and preserved with 1% nitric acid. A separate 2 L plastic bottle was used to collect water samples for chemical parameters. Samples were transported in cooler boxes and analysed within 48 h. Determination of the physical parameters including dissolved oxygen (DO) and temperature were performed onsite using an Aquaread meter from Geowater, Johannesburg, South Africa. An effort was made to maintain collection of samples on the same days monthly which was between the 12th and the 18th of each month for a period of 12 months.

At the laboratory, water samples were filtered through 0.45 micron and analysed for chlorides, fluorides, nitrates and sulfates using ion chromatograph. The unfiltered samples were analysed for TOC using the TOC instrument. The acidified water samples were analysed for selected macro elements (K, Na, Mg and Ca) using ICP–OES. trace metals that included Al, Cu, Co, Fe, Mn, Cr, Cd, Hg, As, Se, Sb, Pb, Ni, Ba, B, U, Sr, and Zn were analysed using ICP–MS. For both instruments, results were exported from the instrument in excel format for further processing.

### Ecotoxicological risk assessment

#### Water quality

The water quality index (WQI) which summarizes the overall quality of water was calculated using Eq. 1, taking into consideration average values of electrical conductivity, pH, TOC, ammonia, chlorides, fluorides, nitrates, sulfates, K, Na, Mg and Ca. The impact of heavy trace metals on water quality was predicted as the Heavy Metal Pollution Index using Eq. 2. Since the Umhlathuze River has agricultural activities, the suitability of its water for irrigation purposes was estimated as the sodium adsorption ratio (SAR) and the Na content using Eq. 3 & 4, respectively. Water is considered suitable for irrigation if SAR < 10 and %Na < 60. Water with SAR > 10 can result in compact and impenetrable soil and %Na > 60 can lead to alkaline or saline soils all of which are not suitable for plant growth (Ali & Ali, 2018).

1 $$WQI =\sum_{i=1}^{n}\left({S}_{i}{W}_{i}\right)$$

2 $$HPI = \frac{{\mathop \sum \nolimits_{i = 1}^{n} \left( {W_{i} \times V_{i} /S_{i} \times 100} \right) }}{{\mathop \sum \nolimits_{i = 1}^{n } \left( {V_{i} /S_{i} \times 100} \right)}}$$

3 $$SAR= \frac{2\times [Na]}{\sqrt{\left[Ca\right]+[Mg]}}$$

4 $$\%Na= \frac{\left[Na\right]+[K]}{\left[Na\right]+\left[K\right]+\left[Mg\right]+[Ca]}$$

where *WQI* is there water quality index, *S*_*i*_ is the standard value, *Wi* is the unit weight of the *i*^*th*^ parameter (ranges from 0 to 1), *n* is the number of parameters. *HPI* is heavy metal pollution index, *V*_*i*_ is the monitored value of *i*^*th*^ parameter. *SAR* is the sodium adsorption ratio.

#### Health risk assessment

Health risk assessment was predicted using three models; the Hazard Index (HI) and incremental lifetime cancer risk (ILCR). The two models give a prediction of the potential health risks to humans due to consumption of contaminated water (Emmanuel et al., 2022; US Environmental Protection Agency, 2011). Firstly, the measured concentrations in water samples were used to estimate the chronic daily intake as an exposure concentration (EC) to a metal per unit body weight per day using Eq. 5. The HI which estimates the cumulative non–carcinogenic risk due to exposure to different metals was calculated as the sum of the ratios of the EC to the reference dose (R_f_D) of each metal using Eq. 6. The ILCR which represents the probability that someone will develop cancer due to continuous exposure over a particular period was calculated as the product of the EC values with cancer slope factor (CSF) values for trace metals with known carcinogenic effect in the body using Eq. 7.

5 $$EC= {C}_{m}\times IR/BW$$

where EC is the exposure concentration (mg kg(bw)^−1^.day^−1^), C_m_ (mg L^−1^) is the concentration of the metal in water, DI is the average daily water intake (2 L day^−1^ for adults, 1 L day^−1^ for children and 0.75 L day^−1^ for infants), BW is the body weight (60 kg for adults, 10 kg for children and 5 kg for infants) (WHO, 2017).

6 $$HI=\sum_{i=1}^{n}\left(EC/{R}_{f}D\right)$$

where HI is the Hazard Index, EC is the exposure concentration (mg/kg(bw) ^−1^.day^−1^), RfD is the reference dosage (mg kg(bw) ^−1^.day^−1^).

7 $$ILCR=\sum_{i=1}^{n}(EC\times CSF)$$

where ILCR is the incremental lifetime cancer risk, EC is the exposure concentration (mg/kg(bw)^−1^ day^−1^) and CSF is the cancer slope factor (mg/kg(bw)^−1^ day^−1^) (Mohammadi et al., 2019).

### Principal component analysis

Principal component analysis (PCA) was done on RStudio statistical software version 4.4.1 (2024-06–14 ucrt) to explore the seasonal interrelationships between elementary levels along Umhlathuze river. The excel results from ICP analysis were converted into a.csv format and imported into RStudio for statistical analysis using the *ggplot* package as the R–environment. The dataset was partitioned such that 80% was used as training data and 20% as testing data. The PCA of the elementary composition was processed using the *prcompt* command. Several PCs were generated but PC1 and PC2 accounted for most of the cumulative variance for the dataset indicating that PC1 vs PC2 could be a reasonable summary of the data set. In this regard, the PCA results were summarized and visualized in terms of biplots of PC1 and PC2 only.

## Results and discussion

### Physicochemical parameters

**Fig. 2:**
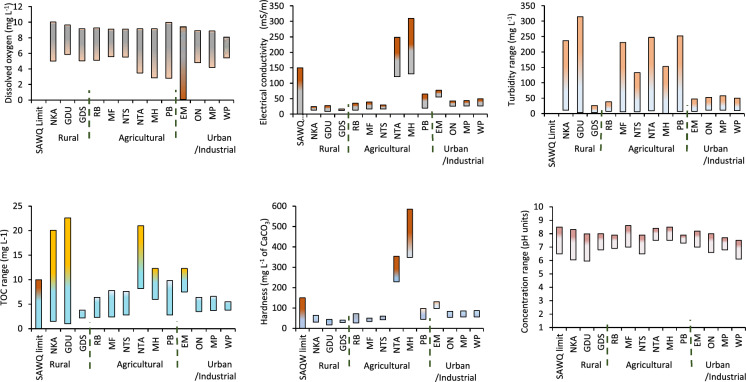
Annual levels of physicochemical parameters

Figure 2 presents the range of physicochemical parameters over a 12 months sampling period from December 2023 to November 2024. Results show moderate seasonal temperature variations ranging between 11.6 and 32.5 °C indicating a warm temperate climate in the region. The temperatures were relatively mild during the winter (May–July) and warm during the summer months. The minimum temperature was observed at the NKA site in the rural area, whilst the maximum was recorded at the MF site in the agricultural area. Water along the Umhlathuze River was generally soft to moderately hard except for the tributaries with Ntambanana (NTA site) and Mholweni (MH site) rivers recording 229–354 and 349–585 mg L^−1^ of CaCO_3_, respectively through the year. Water along Empangeni tributary (EM site) was moderately hard recording an annual range of 98–131 mg L^−1^ of CaCO_3_. Water from the tributaries was therefore not suitable for any domestic purposes.

The DO values ranged from 0.1 to 10 mg L^−1^. The lowest DO was recorded at EM site along Empangeni tributary with the levels below 4 mg L^−1^ for most of the year except in June (Fig. 2). Empangeni River receives wastewater discharge from two municipal wastewater works which could be responsible for the low DO levels. The DO levels below 4 mg L^−1^ have the potential to cause hypoxia in aquatic organisms in some cases leading to death (Zeng et al., 2023). The lower and upper limits for pH were within SAWQ guidelines for all sites except for three instances at the NKA site in July, the GDU site in December and the WP site during the winter months (June and July) where pH values were slightly acidic. Both NKA and GDU sites in the rural areas are located closer to mountains of Babanango and Nkandla, and receive spring water from the region. Spring water pH tends to be slightly acidic due to natural factors including type of rocks and minerals as well as presence of gases like carbon dioxide (Novikov et al., 2022). The relatively low pH value at the Weir Pumpstation (WP site) in the industrial area during the cold dry season could be attributed to reduced dilution effects on industrial effluents during the low flow season.

The electrical conductivity of uMhlathuze River was within the WHO and SAWQ standards throughout the year except for Ntambanana River (NTA site), Mholweni River (MH site) in the agricultural region, and Empangeni River (EM site) in the urban area. This could be attributed to anthropogenic activities including discharge from the quarry (Mholweni River), and agricultural activities (Ntambanana River). Ibe and colleagues conducted a study at Inyibe River of Nigeria, where EC recorded was high and exceeded WHO guidelines. This was due to the impact of discharge from aluminium extrusion activities (Ibe et al., 2019). The EC values along Empangeni River (EM site) were above WHO guidelines ranging between 55 and 77 mS m^−1^ throughout the year.

NTA and MH sites were also observed to have TOC and hardness values above SAWQ guideline limit. The TOC values on Ntambanana River (NTA site) were consistently above guideline limits. Two other sampling points in the rural areas, NKA and GDU sites experienced a spike of TOC measuring about 22 mg L^−1^ in February. The TOC chart for Empangeni tributary (EM site) in the urban and industrial section fluctuated above and below the guideline limit. The river receives wastewater discharge from the local municipal wastewater treatment plants. Other studies elsewhere have identified wastewater discharge as a major contributor of higher TOC values including 36.65 mg L^−1^ on the Nile River in Egypt (Khalil et al., 2023).

Water turbidity in the agricultural region were within 50 NTU most of the time except spikes observed in November and January in the agricultural region and the NKA and GDU sites in the rural areas. These areas have clay soils and the agricultural activities further loosen the soil which get washed into the river during the rainy season. At the urban and industrial sites, turbidity values were below 60 NTU throughout the study due to increase in river level with higher dilution taking place. While most of the turbidity values were relatively low (< 50 NTU), they were still above the SAQW guideline value of 5 NTU indicating a serious challenge for uMhlathuze River.

### Anions

**Fig. 3:**
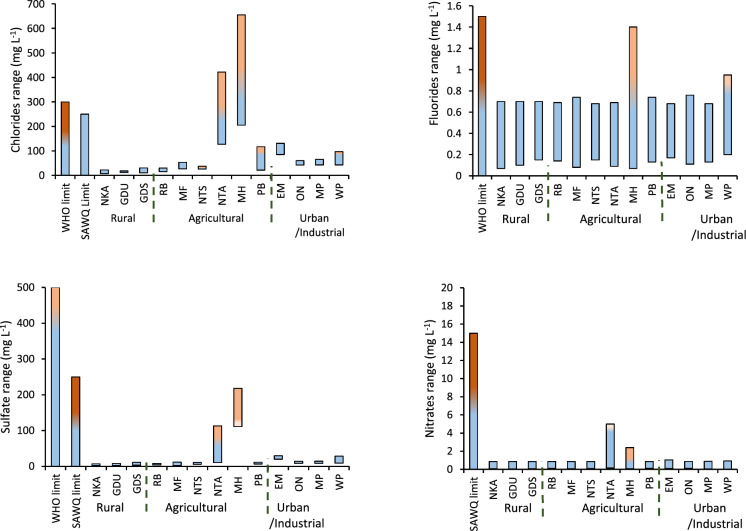
Annual levels for anions for all sampling areas over a 12 month period

The levels of chlorides, fluorides, nitrates and sulfates along Umhlathuze River and its tributaries are summarized in Fig. 3. The results pointed the impact that Ntambanana, Mholweni and Empangeni tributaries have on Umhlathuze River with the three rivers recording extremely high levels compared to other sites. The chloride levels were between 205 and 655 mg L^−1^ for Mholweni River (MH site) with the rainy season (December – March) consistently above the SAQW guideline limits. This could have been due to rain water washing from the quarry excavations contaminating Mholweni River with chlorine–rich rock leachates and chemicals used in blasting operations and lubricants. These levels are similar to those reported on the Achenkovil River Basin where 546 mg L^−1^ of chlorine was recorded (Reghunadh et al., 2023). Both rivers received runoff from quarry mining activities. Ntambanana River (NTA site) which flows mainly through agricultural fields had chlorine levels that ranged from 127 to 422 mg L^−1^. The elevated chloride concentration at this river is probably from natural sources because this area is known for its salty water that has presented challenges for consumers (https://www.news24.com/citypress/voices/democracys–surplus–people–20190321—Last accessed on 23 February 2025). Chloride levels along Empangeni River (EM site) were within guideline limits but ranged from 80 to 141 mg L^−1^ throughout the year.

The elevated chloride levels could be arising from solid waste dumpsite leachates and wastewater plants that discharge their effluent into Empangeni River. The elevated chloride concentrations along the three tributary rivers (MH, NTA and EM sites) had limited impact on the quality of uMhlathuze River because the levels recorded on uMhlathuze River after receiving water from these tributaries was within the guideline limits even though higher compared to levels in the rural areas upstream.

Fluoride levels were within SAWQ guideline limits for all sites. A general trend was observed with the levels relatively higher during the rainy season and recording the lowest levels during the dry cold season (May–July). There were spikes that occurred along the Mholweni River (MH site) reaching 1.4 mg L^−1^ in February. Another spike up to 0.95 mg L^−1^ was recorded at the Weir Pumpstation (WP site) in May. These observations were once off and could not be linked to any source except that Mholweni River emanates from a quarry mining area while the Weir Pumpstation serves as a reservoir for water upstruction purposes. Nitrate levels were also within SAWQ and WHO guideline limits throughout the uMhlathuze River and its tributaries, but the potential impact of quarry activities along Mholweni and farming activities along Ntambanana tributaries were observed with sites slightly higher than other areas along Umhlathuze River.

The sulfate levels were also within guideline limits but elevated levels were observed along the tributaries (NTA and MH sites in the agricultural region, and EM site in the urban and industrial areas. The agricultural activities led to sulfate levels up to 113 mg L^−1^ along Ntambanana River (NTA site) while the quarry activities near Mholweni River (MH site) resulted in sulfate levels ranging between 111 and 218 mg L^−1^. However, these levels were still within the guideline limits. Like chlorides, sulfates can also be linked to the type of soil in that particular geographical area, but fertilizer runoff from agricultural fields is also a major contributor (Saka et al., 2024; Sukarjo et al., 2025). A general increase in sulfate levels downstream of the uMhlathuze River was observed with the urban and industrial segment recording high levels compared to rural and agricultural segments. The levels in the rural areas were consistently below 10 mg L^−1^, the agricultural segment had about 10 mg L^−1^, while the urban and industrial segment were consistently above 10 mg L^−1^, which could be suggestive of cumulative effects down the river. Accumulation of sulfates in the industrial area (WP site) could also be directly from the industries within industrial area or the result of sea spray since the Weir Pumpstation is closest to the Indian ocean.

### Elementary analysis

#### Macro elements, (major cations)

**Fig. 4:**
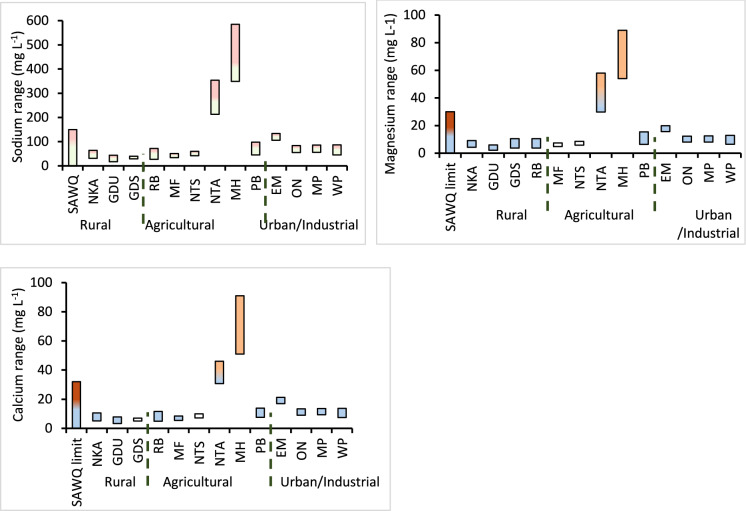
Annual levels of sodium, magnesium and calcium along the uMhlathuze River

The concentration range of macro elements for the 12 months period are summarised in Table 1. The levels recorded for each month are given on the Supplementary. Potassium was within the SAQW permissible limits throughout the year for all sampling sites. However, Ca, Mg and Na were recorded above the guideline limits for the Ntambanana (NTA site) and Mholweni (MH site) tributaries throughout the year (Fig. 4). These elevated levels could be linked to quarry works along the Mholweni River and the geochemistry of the area where Ntambanana River emanates. The impact of similar anthropogenic activities on the levels of macro elements in river water including silicate weathering, dissolution, leaching, and infiltration from hard rock and soil quarries has been reported in India (Reghunadh et al., 2023) and Kenya (Chebet et al., 2020). The impact of elevated levels of macro elements from MH and NTA sites is minimal on UMhlathuze River because the levels recorded at PB site along uMhlathuze River after it has received water from the two streams were within guideline values suggesting dilution effects due to larger volumes of water along uMhlathuze River. Despite the dilution effects, the levels of macro elements were relatively higher in the urban and industrial area with Empangeni River (EM site) also pumping more macro elements into Umhlathuze River. The relatively high levels along Empangeni tributary might have been caused by wastewater discharge from local wastewater works and possible runoff form Empangeni town and surrounding informal settlements.

**Table 1:** Minimum, maximum and median values for non–toxic elements over 12 months

Segment	Site	Concentration of macro elements (mg L^−1^)				Concentration of metals (ug L^−1^)											
		Ca	Mg	Na	K	Al	Ba	B	Sr	Sb	Se	Cu	Ni	Fe	Mn	Zn	Co
Rural	NKA	5.2–11	4.3–9.1	11–18	0.8–2.6	3.2–2463	19.5–259	8.4–43.5	37.1–99	nd–0.5	nd–9.4	0.7–5.0	0.3–21.7	17.4–2155	1.0–713	1.5–29	nd–2.5
	GDU	3.3–7.9	2.1–5.9	11–16	0.8–2.4	4.5–1985	16.4–140	8.3–81	29.4–104	nd –2.1	nd–9.8	0.3–69.9	0.2–38.8	6.0–3894	0.4–633	0.1–59	nd–6.7
	GDS	5.1–7.1	3.7–11	11–17	1.5–2.1	33.7–1191	27.9–46.6	14.7–24.1	36.5–54	nd –0.4	nd–8.4	0.4–7.0	0.6–27.8	138.4–880	0.8–32	0.6–5.2	nd –0.7
Agricultural	RB	4.9–12	3.7–11	14–48	1.5–2.7	2.5–1318	0.6–66	3.6–56	0.7–100	nd –0.4	nd –8.0	0.6–4.6	0.5–28.1	1.2–1256	0.7–69	nd–7.1	nd –1.2
	MF	5.5–8.5	4.8–7.4	24–32	1.5–3	nd–1173	1.9–128	nd–171	2.0–309	nd –1.8	nd –9.8	1.2–5.5	0.3–20.3	0.7–2298	1.3–158	nd–4.4	nd –1.7
	NTS	7.2–10	5.8–8.5	22–40	1.7–3.1	nd–1357	0.9–92	nd–55	0.9–86	nd –0.8	nd –13.8	0.3–3.1	0.3–29.4	0.1–1967	0.2–209	nd–3.0	nd –1.0
	NTA	31–46	30–58	189–554	1.2–8.4	nd–2033	38.1–281	24.3–216	40–435	nd –3.5	nd –20.7	0.8–5.1	0.3–33.8	0.1–2160	0.3–539	0.1–5.5	nd –5.3
	MH	51–91	54–89	179–552	1.7–3.5	nd–1836	8.8–233	nd–228	8.8–823	nd –7.4	nd –19.4	nd–8.6	0.2–59.9	nd–2583	0.1–260	nd–16.9	nd –9.1
	PB	7.6–14	6.3–15	26–92	1.6–3.2	32.7–1713	34.2–168	28.6–80	58.7–147	nd –0.8	nd –26.6	0.8–4.4	0.6–33.5	145–2143	1.5–268	1.2–10.0	nd –2.3
Urban and industrial	EM	17–21	16–20	78–101	4.3–6.9	5.3–695	nd–92	63.5–83	1.2–179	nd –1.2	nd –27.8	0.7–6.2	0.9–15.8	1.5–1347	0.1–177	nd–234	nd –1.2
	ON	9–13	8–12	34–52	2–3.4	2.3–1892	nd–77	2.8–69	0.9–117	nd –0.6	nd –23.2	0.4–11.6	0.4–26.2	1.1–1688	0.1–205	nd–427	nd –2.0
	MP	9.4–14	8–13	33–54	2.3–3.2	7.8–2206	36.7–78	34.9–72	53.1–121	nd –0.2	nd –27.9	0.4–6.3	0.4–24.9	14.2–2507	0.1–159	nd–6.0	nd –2.0
	WP	5.5–7.5	6.4–13	28–54	2.2–3.4	nd–436	0.6–72	4.3–55	nd–134	nd –1.4	nd –26.7	nd–3.7	nd–7.2	1.4–2317	nd–142	nd–8.1	nd –1.2
WHO guidelines		–	–	–	900*	200	1300	–	–	40	40	2000	2000*	400*	3000*	–	
SAWQ Guideline	32	32	200	200													

*WHO health–based precautional limit

#### Levels of trace essential elements

The annual trends of trace essential elements are summarized in Table 1. The concentration levels recorded for each month over the 12–month period are given on the Supplementary. The levels of Cu, Ni and Zn were within the WHO permissible limits at all sites throughout the year, while Fe and Mn at times exceeded their WHO precautional limits during the rainy season. There are no WHO guideline limits for Co, but in the current study its levels were mostly within 2 µg L^−1^ except for the MH site along Mholweni River and single spikes on the GDU and NTA sites in November. A significant spike in Zn levels was observed at the EM and ON sites during the month of December recording 234 and 427 µg L^−1^, respectively while the other sites were consistently below 60 µg L^−1^. The accumulation of Zn on Empangeni River (EM site) could be due to wastewater effluents and direct runoff from activities and structures that use galvanized steel structures within Empangeni urban area. Consequently, the spike at the ON site could be due to accumulation from Empangeni River since it occurs downstream after uMhlathuze River has met with the Empangeni River. These spikes may have indicated effects of the start of the rainy season but the impact on quality of uMhlathuze River was minimal with the levels still within the WHO permissible limits. The levels of Ni were within WHO guideline limits and normal drinking levels (< 20 µg L^−1^) throughout the year (WHO, 2017), but significant spikes were observed for all sites during the beginning of the rainy season in November. The spikes were much significant along Mholweni River (MH site) increasing up to 60 µg L^−1^. Mholweni River has quarry activities which could potentially result in leaching of Ni into the river system.

WHO has no guideline limits for Fe and Mn but has set precautional values of 2000 and 400 µg L^−1^, respectively (WHO, 2017). The Fe levels were relatively high but within the 2000 µg L^−1^ limit except for a few isolated instances during the rainy season where this limit was exceeded. The area around uMhlathuze River has brownish soils and iron oxide together with organic matter are responsible for this colour which might explain the high levels of Fe in the river. As rainwater infiltrates the soil and underlying geological formations, it can leach Fe to rivers as part of surface run–off (Conrad et al., 2020; Tashiro et al., 2020). Other studies have observed elevated levels of Fe in rivers due to the geochemistry of the region through which the river passes (Aleshina et al., 2024; Vodyanitskii et al., 2018). Besides the spike that occurred in November at the GDU site, the levels of Fe in the rural section of uMhlathuze River were generally lower with the levels continuously increasing downstream. Elevated levels of Fe due to anthropogenic activities have recently been reported in other rivers within South Africa including the Vaal River Basin (Moloi et al., 2020), Nzhelele River (Edokpayi et al., 2017), the Kaap River (Maphanga et al., 2024) and Mthatha River (Madikizela et al., 2023), while lower levels have been recorded elsewhere including Mohlapitsi River (Addo–Bediako, 2024).

The concentration levels for Mn were also within the WHO health–based precautional limit (400 µg L^−1^) for most of the year except for isolated spikes observed at NKA, GDU and NTA during November which marks the beginning of the rainy season. The spike in the rural areas was huge increasing from < 100 µg L^−1^ up to 633 and 713 µg L^−1^ at GDU and NKA sites, respectively. Besides the few spikes observed in the rural and agricultural sections, the Mn levels were generally higher in the urban and industrial section of the uMhlathuze River. With no evidence of the impact of wastewater effluents on the EM site along Empangeni River, the Mn levels could have emanated from local industries within the urban and industrial sections of the study. Industrial areas have been reported previously to elevate levels of Mn in river systems.

#### Other trace metals

There are no guideline limits for Al but a health–based precautional value of 900 µg/L is recognized (WHO, 2017). It was observed that concentration levels of Al during the rainy season from (November*–*March) were consistently above this value except for Empangeni tributary (EM site) and the Weir Pumpstation (WP) in the urban and agricultural region. The high levels observed in the rural areas could not be linked to any anthropogenic activities. Probably, during heavy rains leachable Al from the geology of Babanango and Nkandla may be washed into Umhlathuze River. Downstream of the river, Al could arise from agricultural, urban and industrial activities. This was mentioned by Mthembu and colleagues in their study at the same river in 2012, where Al was reported in higher concentrations at the ON, MP and WP sites (Mthembu et al., 2012). Elsewhere, Al has been reported in extremely high levels of 1.172*–*29.094 mg L^−1^ (Edokpayi et al., 2017).

The levels of B and Ba were within WHO permissible limits for all sites throughout the year with a few spikes observed in the rainy season along Mholweni (MH site) and Ntambanana (NTA site) and Empangeni (EM site) tributaries. The levels of B along Empangeni River (EM site) were also a bit elevated compared to other sites ranging between 60 and 80 µg/L throughout the year. The levels of Se were within WHO permissible levels throughout the year. The Sb levels were mostly around 0.2 µg L^−1^ which is normal for natural unpolluted even though a few spikes up to 7.4 and 3.5 µg L^−1^ for MH and NTA sites, respectively were observed. The Sr levels were within 100 µg L^−1^ for most parts of the year except for MH, NTA and EM sites which represent the tributaries to Umhlathuze River. The seasonal concentrations recorded for each month are given on the Supplementary.

### Trace toxic elements

**Fig. 5:**
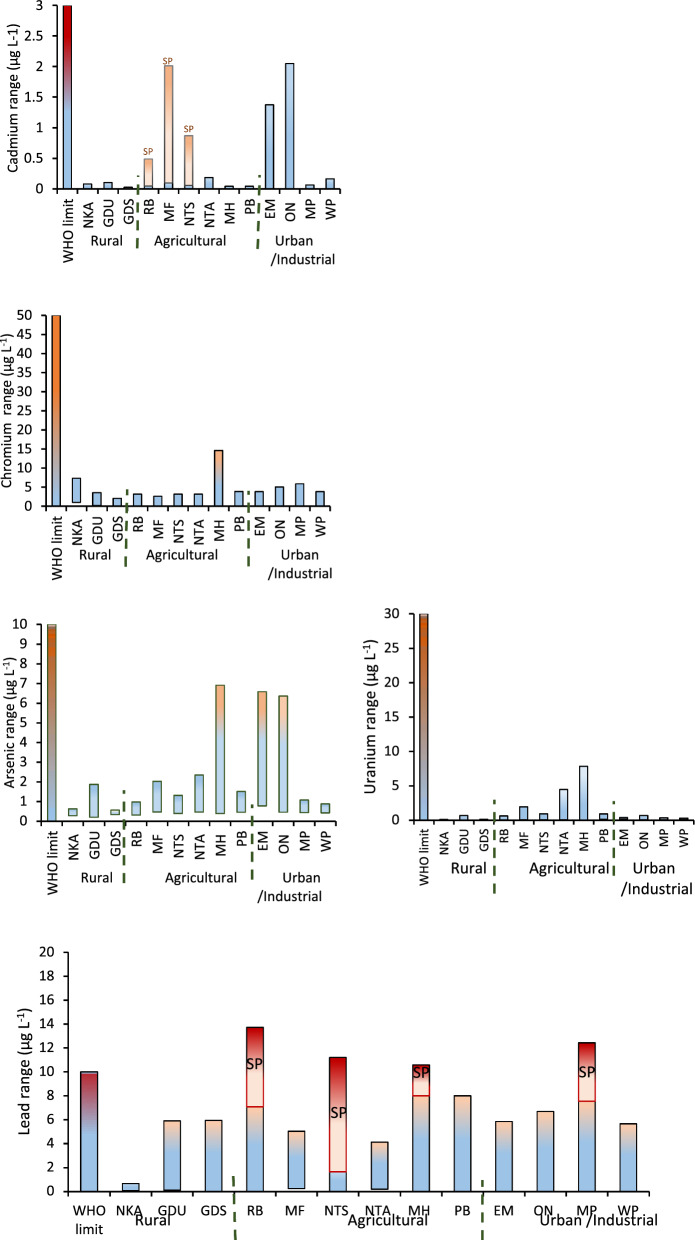
Concentration range for trace toxic metals over the 12–months period. SP, single spike

Figure 5 represents the concentration range for the trace toxic elements (Cd, Cr, As, Pb and U) over a 12 months sampling period from December 2023 to November 2024. The seasonal concentrations recorded for each month are given on the Supplementary. The results show that the levels of Cd, Cr, As, U and Hg were all within the WHO permissible limits. Mercury was not detected except along the Empangeni tributary (EM site) where 0.399 µg L^−1^ was recorded along Empangeni River (EM site) in the month of March. This falls within the WHO permissible limit of 6 ug/L (WHO, 2017). Traces of Hg were also detected at the ON site which occurs after the Empangeni–Umhlathuze juncture recording 0.014 and 0.033 µg L^−1^ in the months of January and June, respectively. Traces of Hg were also detected on the Mholweni tributary in March (0.112 µg L^−1^) and along the Ntambanana tributary (0.017 µg L^−1^) in the month of May. Mthembu and colleagues from their study at the same river also reported Hg concentration levels within guideline limits (Mthembu et al., 2012).

The levels of Pb were within the WHO permissible limits for most of the year (nd–8 µg L^−1^), but there were a few isolated incidents where the levels were above the guideline limits. For example, in May the RB site in the agricultural region recorded 13.7 µg L^−1^, while the MP site in the industrial area recorded 12.4 µg L^−1^. In March, the NTS site recorded 11.02 µg L^−1^ while the MH site affected by quarry activities recorded 10.6 µg L^−1^. These were isolated incidents and could have resulted from episodic incidents within this region. Similar levels have been reported along Nzhelele River where concentration varied from 1 and 13 µg L^−1^ (Edokpayi et al., 2017) while at Mutangwi River the Pb levels were above 50 µg L^−1^ (Madilonga et al., 2021). A previous study conducted by Mthembu and colleagues reported 280, 380 and 500 µg L^−1^ Pb at ON, MP and WP sites, respectively which exceeded guideline limits and higher than the levels reported in the current study (Mthembu et al., 2012).

The Cd concentrations were very low (< 0.09 µg L^−1^) in the rural and agricultural areas except for the once–off spikes that occurred in March at the RB site (0.5 µg L^−1^), MF (2 µg L^−1^) and NTS (0.9 µg L^−1^). The spikes were still within the guideline limits but they might indicate the potential impact of runoff from fertilized farms in the area. In the urban and industrial area, the Cd levels were relatively high along Empangeni River (EM) that emerges from Empangeni Town, which could point to wastewater effluents and urban runoff as a source of Cd into Umhlathuze River. The levels along Empangeni River led to higher levels recorded on the ON site that represents a point after the Empangeni–Umhlathuze intersection. The impact is however minimal, with the levels still within guideline limits. Mthembu and colleagues previously reported 4 µg L^−1^ Cd at the WP site which was above permissible limits (Mthembu et al., 2012). Fifteen years later, this study indicates no threat to water quality due to Cd concentration levels at the same sites and the rest of uMhlathuze River sites upstream.

Figure 5 shows that As levels along Umhlathuze River were within the levels expected for natural water which is less than 1–2 µg L^−1^ (WHO, 2017). However, the impact of urban activities including domestic wastewater effluents was evident with Empangeni River (EM site) recording between 0.779 and 6.58 µg L^−1^ throughout the year. The highest concentrations were recorded in November and January reaching 1.59, 6.58 and 1.70 µg L^−1^, respectively. Likewise, this had an impact on the ON site which also recorded the corresponding levels of 1.01, 6.37 and 0.730 µg L^−1^, respectively for the same months. It was also observed that the As levels along Mholweni river (MH site) were affected by quarry activities resulting in concentrations of 0.393 to 6.92 µg L^−1^ throughout the year (Fig. 5). Importantly, the impact of these activities was still minimal because the levels were within the guideline limits.

### Elementary interrelationships

**Fig. 6:**
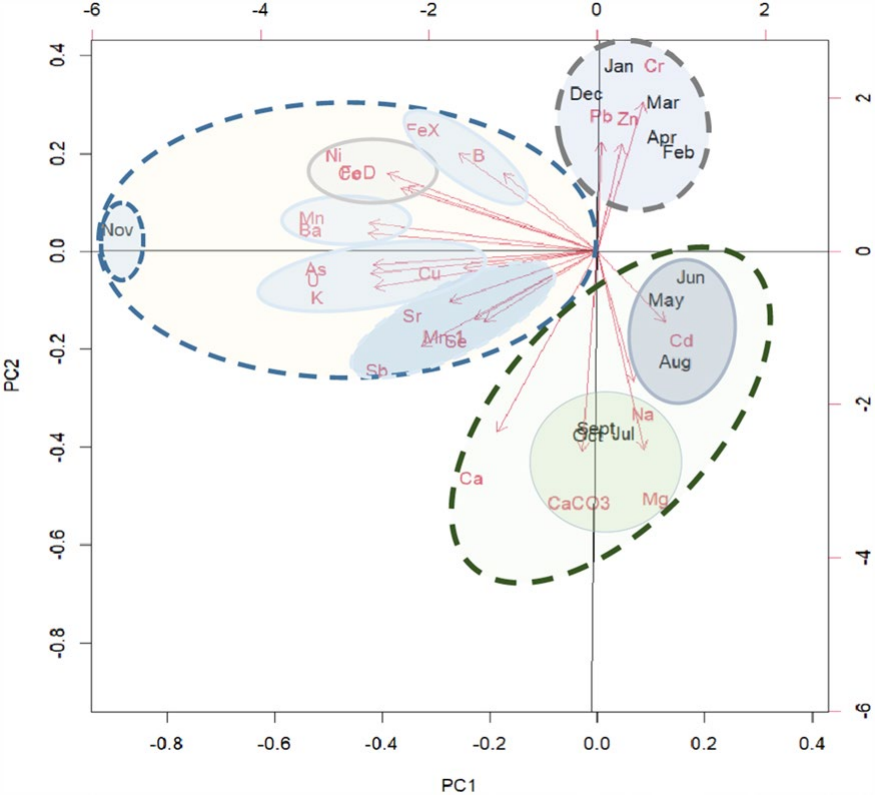
Interrelationships between elementary levels along Umhlathuze River over a 12–month period

A summary of the relationships between seasonal levels of metals along Umhlathuze River is presented as a biplot in Fig. 6. PC1 was the most important dimension that captured the largest amount of variation in the levels of metals. In this regard, the plot revealed three distinct seasonal groupings. The months of December–April which represent the rainy season were clustered together and defined by higher levels of Pb, Zn and Cr. This interrelationship between Pb, Zn and Cr could suggest a common source for these metals that is brought to the river by surface runoff during the high flow season. Previous studies on urban river pollution have observed a similar correlation between these metals and identified corrosion of galvanized steel structures from small–scale industries and informal dwellings that make their way into the environment as part of urban runoff as the probably source (Madikizela et al., 2023).

Another distinct cluster occurred from May–September primarily influenced by macro elements as well as Cd. A strong correlation occurs among macro elements suggesting that they were influenced by the low flow along the river. This could suggest that their natural existence in the river overweighs the influx from surface runoff and therefore become concentrated as the river water levels reduce during the dry winter/spring season. Most studies have found Cd interrelated with other trace elements. Cadmium was not detected in most of the months which could explain its deviation from other trace metals. Other studies have also observed that Cd trends were not corelated with other trace metals (Madikizela et al., 2023).

The final seasonal grouping was clustered in November consisting of most of the trace metals with a surprise of K which is a macro element. November marks the beginning of the rainy season and the high levels of these trace elements could result from the first flush effect where the first rains wash and accumulate the trace metals into the river after a dry spell (Conrad et al., 2020). While most of these elements were within the guideline limits, elevated levels along the tributaries were linked to wastewater effluents and urban runoff (EM site), quarry activities (MH site) and the geochemistry and agricultural activities (mainly NTA site). The levels of these elements remained high throughout the rainy season gradually decreasing until reaching minimum in June/July. Unlike the macro elements, the observation about trace elements maximizing during the rainy season suggests that the rains accumulate the trace elements into the river as part of surface runoff. Within this group, there were various definite clusters that showed close correlations between certain trace metals. For example, B is known to adsorb on iron oxides and expectedly its trend showed a strong relationship with Fe (Demetriou & Pashalidis, 2012).

### Risk assessment

#### Water quality assessment

Table 2 represents WQI for uMhlathuze River in rural, agricultural, urban and industrial areas. Most of the sites had WQI < 20 and therefore were rated in the excellent category. Besides elevated concentration levels for physicochemical parameters as well as those from macro elements, the WQI values for NTA, MH and EM sites were still acceptable and rated as good. The HPI values calculated based on trace toxic metal concentrations are also summarized in Table 2. Overall HPI values were very low for all sites implying that the anthropogenic activities including agricultural and industrial development had minimal potential to cause pollution concerns due to toxic metals on Umhlathuze River.

**Table 2:** Water quality and health risk assessment parameters for Umhlathuze River

Segment	Site	Water quality indices				Suitability for irrigation			Hazard index (HI) value				Cancer risk (ILCR value × 10^−4^)		
		WQI	Rating	HPI	Limit	SAR	Rating	Na (%)	Rating	Adult	Child	Infant	Adult	Child	Infant
Rural area	NKA	18.8	Excellent	6	100	7.6	Excellent	52	Permissible	0.29	0.88	1.32	1.86	5.59	8.38
	GDU	15.8	Excellent	9	100	8.3	Excellent	59	Permissible	0.32	0.97	1.46	1.71	5.13	7.70
	GDS	10.7	Excellent	5	100	9.2	Excellent	62	Doubtful	0.16	0.47	0.71	1.41	4.23	6.35
Agricultural	RB	15.6	Excellent	6	100	10.8	Good	62	Doubtful	0.24	0.72	1.07	1.60	4.81	7.21
	MF	17.8	Excellent	8	100	15.5	Good	70	Doubtful	0.37	1.12	1.68	1.62	4.87	7.30
	NTS	15.4	Excellent	6	100	13.4	Good	65	Doubtful	0.27	0.80	1.19	1.66	4.98	7.47
	NTA	30.2	Good	7	100	76.3	Unsuitable	81	Unsuitable	0.47	1.42	2.13	2.19	6.56	9.83
	MH	36.1	Good	9	100	44.2	Unsuitable	69	Doubtful	0.73	2.18	3.28	4.99	15.0	22.4
	PB	20.2	Excellent	6	100	17.2	Good	68	Doubtful	0.27	0.82	1.23	1.88	5.65	8.47
Urban and Industrial	EM	26.0	Good	9	100	29.5	Unsuitable	72	Doubtful	0.48	1.43	2.15	2.23	6.68	10.0
	ON	20.4	Excellent	10	100	17.5	Good	67	Doubtful	0.48	1.43	2.15	2.15	6.46	9.70
	MP	18.1	Excellent	6	100	18.2	Permissible	67	Doubtful	0.34	1.03	1.54	2.21	6.62	9.93
	WP	18.7	Excellent	7	100	18.0	Permissible	67	Doubtful	0.26	0.79	1.18	1.20	3.60	5.39

WQI, water quality index; HPI, heavy metal pollution index; SAR, sodium adsorption ratio; HI, hazard index; TCR, total cancer risk

The water quality in relation to irrigation was excellent in the rural areas with the SAR values ranging between 7.6 and 9.2. The river water was still good for irrigation in the agricultural region with SAR values ranging between 10.8 and 17.2, but water along the two tributaries (Mholweni and Ntambanana Rivers) was deemed unsuitable with the potential to harden the soil and reduce internal drainage. In the urban and industrial areas, Empangeni River water was deemed unsuitable with a SAR value of 27.8 while the water along Umhlathuze River was found to be suitable. The Na content as a %Na value was up to 81% for Ntambanana River and its water was deemed unsuitable for irrigation. This value together with the high chlorine levels reported in Sect. 3.2 indicated that the water from Ntambanana River was likely to be too salty for irrigation purposes. It is important to note that SAR and %Na values are not an indication of pollution but an estimation of water impermeability and saltness which is might hinder plant growth. Other studies have reported similar observations where some parts of a river were unsuitable for irrigation purposes including Molo River in Kenya (Chebet et al., 2020). The impact of tributaries on the main river quality has also been reported in literature including the Pechora River in Russia (Yakovlev et al., 2023).

#### Hazard index and incremental lifetime cancer risk

The HI values based on non–carcinogenic effects and ILCR values based on carcinogenic effects within uMhlathuze River are summarized in Table 2. While there is no evidence of using the raw water from Umhlathuze River for consumption purposes, it was still important to assess the potential risk considering the that river stretches from rural areas where access to treated water may be a challenge. In this regard, the results in Table 2 indicate that water from Umhlathuze River will not cause any health problems related to metal contamination with HI values below 1 for all sites. These values do not take into consideration other pollutants that may enter the river from wastewater treatment plants. In case of children, the water in the rural areas (NKA, GDU and GDS sites) was safe while other regions were not safe especially along the three tributaries (MH, NTA and EM sites) whose HI values were between 1.42 and 2.18. It is important to note that it is unlikely that people can drink the unprocessed water from these tributaries. Finally, the untreated river water was found to be unsuitable for consumption by infants throughout the entire sampling region except for the GDU site whose predicted HI value was 0.71.

## Conclusions and recommendations

The study has provided insights on the potential impact of anthropogenic activities on the water quality along Umhlathuze River and its three main tributaries based on levels of physicochemical properties, anions, macro elements and trace metals. Along the main river, these parameters were mostly within guideline limits but they were elevated and at times above the guideline limits along the tributaries. Agricultural activities, quarrying, wastewater effluents and urban runoff were identified as potential pollution sources along the tributaries. However, a crucial finding is that the elevated pollution levels along the tributaries had minimal impact on Umhlathuze River with the parameters immediately diluted to normal conditions as soon as the water from the tributaries entered the main river. Despite this resilience of Umhlathuze River, dependency on its dilution capabilities cannot be a sustainable management strategy. The tributaries act as critical and chronic pollution hotspots that should be closely monitored to prevent the river from being overwhelmed on the long run.

The study further demonstrated that water quality along Umhlathuze River and its tributaries is affected by seasonality and anthropogenic activities within its tributaries. Based on principal component analysis, the elementary levels along Umhlathuze River and its tributaries were grouped into three distinct groups; the rainy season (December*–*April) cluster consisting of trace metals associated with surface runoff; the dry season (May*–*September) cluster characterized by macro elements that tend to concentrate during the cold low flow season; and the unique first flush event in November marked by those trace elements that experience a spike during the first rains. This insight may be crucial in providing a basis for source control and a more dynamic monitoring schedule that targets pollution sources and the potential impact of episodic pollution spikes along Umhlathuze River and its tributaries. With the study focusing on the aqueous phase of the river, further studies can be done to include the sediments and microorganisms that exist along Umhlathuze River.

## Supplementary Information

Below is the link to the electronic supplementary material.

Supplementary file1 (XLSX 84 KB)

## Data Availability

No datasets were generated or analysed during the current study.

## References

[CR1] Addo-Bediako, A. (2024). Spatiotemporal assessment of heavy metal(loid) pollution in water and surface sediment of the Mohlapitsi River, South Africa. *Polish Journal of Environmental Studies,**33*(4), 3545–3554. 10.15244/pjoes/169451

[CR2] Aleshina, A., Rusakova, M. A., Drozdova, O. Y., Pokrovsky, O. S., & Lapitskiy, S. A. (2024). Dissolved iron and organic matter in boreal rivers across a South-North transect. *Environments,**11*(4), 1–13. 10.3390/environments11040065

[CR3] Ali, S. A., & Ali, U. (2018). Hydrochemical characteristics and spatial analysis of groundwater quality in parts of Bundelkhand Massif. *India. Applied Water Science,**8*(1), 1–15. 10.1007/s13201-018-0678-x

[CR4] Chebet, E. B., Kibet, J. K., & Mbui, D. (2020). The assessment of water quality in river Molo water basin, Kenya. *Applied Water Science,**10*(4), 1–10. 10.1007/s13201-020-1173-8

[CR5] Conrad, S. R., Santos, I. R., White, S. A., Hessey, S., & Sanders, C. J. (2020). Elevated dissolved heavy metal discharge following rainfall downstream of intensive horticulture. *Applied Geochemistry,**113*(December 2019), 104490. 10.1016/j.apgeochem.2019.104490

[CR6] Demetriou, A., & Pashalidis, I. (2012). Adsorption of boron on iron-oxide in aqueous solutions. *Desalination and Water Treatment,**37*(1–3), 315–320. 10.1080/19443994.2012.661288

[CR7] Dlamini, S., Nhleko, B., & Ubisi, N. (2024). Understanding socioeconomic risk and vulnerability to climate change–induced disasters: The case of informal settlements in KwaZulu–Natal, South Africa. *Journal of Asian and African Studies*, 1–20. 10.1177/00219096241275398

[CR8] Dzhangi, T. R., & Atangana, E. (2024). Evaluation of the impact of coal mining on surface water in the Boesmanspruit, Mpumalanga, South Africa. *Environmental Earth Sciences,**83*(6), 1–21. 10.1007/s12665-024-11431-6

[CR9] Edokpayi, J. N., Odiyo, J. O., Popoola, O. E., & Msagati, T. A. M. (2016). Assessment of trace metals contamination of surface water and sediment: A case study of Mvudi river, South Africa. *Sustainability (Switzerland),**8*(2), 1–13. 10.3390/su8020135

[CR10] Edokpayi, J. N., Odiyo, J. O., Popoola, E. O., & Msagati, T. A. M. (2017). Evaluation of temporary seasonal variation of heavy metals and their potential ecological risk in Nzhelele River, South Africa. *Open Chemistry,**15*(1), 272–282. 10.1515/chem-2017-0033

[CR11] Emmanuel, U. C., Chukwudi, M. I., Monday, S. S., & Anthony, A. I. (2022). Human health risk assessment of heavy metals in drinking water sources in three senatorial districts of Anambra State, Nigeria. *Toxicology Reports,**9*, 869–875. 10.1016/j.toxrep.2022.04.011 36518376 10.1016/j.toxrep.2022.04.011 PMC9742819

[CR12] Harding, G., Chivavava, J., & Lewis, A. E. (2020). Challenges and shortcomings in current South African industrial wastewater quality characterisation. *Water SA,**46*(2), 267–277. 10.17159/wsa/2020.v46.i2.8242

[CR13] Ibe, F. C., Opara, A. I., Ibe, B. O., & Amaobi, C. E. (2019). Application of assessment models for pollution and health risk from effluent discharge into a tropical stream: Case study of Inyishi River, Southeastern Nigeria. *Environmental Monitoring and Assessment*. 10.1007/s10661-019-7936-8 10.1007/s10661-019-7936-8 31734747

[CR14] Jomova, K., Alomar, S. Y., Nepovimova, E., Kuca, K., & Valko, M. (2024). Heavy metals: Toxicity and human health effects. *Archives of Toxicology*. 10.1007/s00204-024-03903-2 10.1007/s00204-024-03903-2 PMC11742009 39567405

[CR15] Khalil, M. M., Aboueldahab, S. M., Abdel–Raheem, K. H. M., Ahmed, M., Ahmed, M. S., & Abdelhady, A. A. (2023). Mixed agricultural, industrial, and domestic drainage water discharge poses a massive strain on freshwater ecosystems: A case from the Nile River in Upper Egypt. *Environmental Science and Pollution Research International,**30*(58), 122642–122662. 10.1007/s11356-023-30994-8 37973780 10.1007/s11356-023-30994-8

[CR16] Madikizela, L. M., Chimuka, L., & Ncube, S. (2023). Metal pollution source apportionment in two important rivers of Eastern Cape Province, South Africa: A case study of Bizana and Mthatha Rivers. *Environmental Forensics,**24*(1–2), 71–84. 10.1080/15275922.2021.1940382

[CR17] Madilonga, R. T., Edokpayi, J. N., Volenzo, E. T., Durowoju, O. S., & Odiyo, J. O. (2021). Water quality assessment and evaluation of human health risk in Mutangwi river, Limpopo province, South Africa. *International Journal of Environmental Research and Public Health*. 10.3390/ijerph18136765 10.3390/ijerph18136765 PMC8296923 34202418

[CR18] Maphanga, T., Chidi, B. S., Phungela, T. T., Gqomfa, B., Madonsela, B. S., Malakane, K. C., et al. (2024). The interplay between temporal and seasonal distribution of heavy metals and physiochemical properties in Kaap River. *International Journal of Environmental Science and Technology,**21*(7), 6053–6064. 10.1007/s13762-023-05401-x

[CR19] Mashao, F. M., Mothapo, M. C., Munyai, R. B., Letsoalo, J. M., Mbokodo, I. L., Muofhe, T. P., et al. (2023). Extreme rainfall and flood risk prediction over the east coast of South Africa. *Water (Switzerland),**15*(1), 1–19. 10.3390/w15010050

[CR20] Mohammadi, A. A., Zarei, A., Majidi, S., Ghaderpoury, A., Hashempour, Y., Saghi, M. H., et al. (2019). Carcinogenic and non-carcinogenic health risk assessment of heavy metals in drinking water of Khorramabad, Iran. *MethodsX,**6*, 1642–1651. 10.1016/j.mex.2019.07.017 31372352 10.1016/j.mex.2019.07.017 PMC6660555

[CR21] Mokoma, T. J., & Tilahun, S. L. (2022). Water provision planning on the basis of human population growth forecasts: A case study of the City of uMhlathuze, KwaZulu-Natal Province, South Africa. *Water Supply,**22*(9), 7172–7188. 10.2166/ws.2022.290

[CR22] Moloi, M., Ogbeide, O., & Voua Otomo, P. (2020). Probabilistic health risk assessment of heavy metals at wastewater discharge points within the Vaal River Basin, South Africa. *International Journal of Hygiene and Environmental Health,**224*(October 2019), 113421. 10.1016/j.ijheh.2019.113421 31784328 10.1016/j.ijheh.2019.113421

[CR23] Mthembu, M. S., Djarova, T. G., & Basson, A. K. (2012). Effect of agricultural and industrial developments on the quality of water at UMhlathuze River (Northern Coast of Kwa–Zulu Natal, RSA). *African Journal of Microbiology Research,**6*(9), 2020–2026. 10.5897/ajmr11.1227

[CR24] Novikov, A. I., Shirokaya, A. A., & Slukovskaya, M. V. (2022). Elemental concentrations of major and trace elements in the spring waters of the arctic region of Russia. *Minerals*, *12*(1). 10.3390/min12010008

[CR25] Ntombela, C., Funke, N., Meissner, R., Steyn, M., & Masangane, W. (2016). A critical look at South Africa’s green drop programme. *Water SA,**42*(4), 703–710. 10.4314/wsa.v42i4.21

[CR26] Reghunadh, K., Antony, S., Arun, V., Krishnakumar, A., Abhirami, J. S., Shehna, S., et al. (2023). Impact of stone quarries on groundwater quality at Achenkovil River Basin, Southern Western Ghats, India: Investigation using WQI and GIS. *Environmental Quality Management,**33*(2), 325–341. 10.1002/tqem.22079

[CR27] Saka, D., Antwi, E. O., Skrzypek, G., Adu-Gyamfi, J., Heng, L., & Attiogbe, F. (2024). Tracing sulfate sources in a tropical agricultural catchment with a stable isotope Bayesian mixing model. *Science of the Total Environment,**951*(June), 175502. 10.1016/j.scitotenv.2024.175502 39147051 10.1016/j.scitotenv.2024.175502

[CR28] Sukarjo, Yustika, R. D., Handayani, C. O., Dewi, T., Yustiawati, Yanti, D., & Dariah, A. (2025). Risk assessment for non–carcinogenic effect posed by sulfates in water on the health of residents around The Sumpur River, West Sumatra–Indonesia. *Toxicology Reports*, *14*(January). 10.1016/j.toxrep.2025.101921 10.1016/j.toxrep.2025.101921 PMC11803871 39926411

[CR29] Tashiro, Y., Yoh, M., Shiraiwa, T., Onishi, T., Shesterkin, V., & Kim, V. (2020). Seasonal variations of dissolved iron concentration in active layer and rivers in permafrost areas. *Russian Far East. Water (Switzerland),**12*(9), 1–17. 10.3390/W12092579

[CR30] US Environmental Protection Agency. (2011). Exposure Factors Handbook: 2011 Edition. *U.S. Environmental Protection Agency*, *EPA/600/R*–(September), pp. 1–1466. EPA/600/R-090/052F

[CR31] van Rensburg, S. J., Barnard, S., & Krüger, M. (2016). Challenges in the potable water industry due to changes in source water quality: Case study of Midvaal Water Company, South Africa. *Water SA,**42*(4), 633–640. 10.4314/wsa.v42i4.14

[CR32] Vodyanitskii, Y. N., Kirillova, N. P., Manakhov, D. V., & Karpukhin, M. M. (2018). Iron compounds and the color of soils in the Sakhalin Island. *Eurasian Soil Science,**51*(2), 163–175. 10.1134/S1064229318020138

[CR33] WHO. (2017). *Guidelines for drinking water quality: 4th ed, Incorporating the first addendum*. 10.5005/jp/books/11431_8 28759192

[CR34] Yakovlev, E., Druzhinin, S., Druzhinina, A., Zykov, S., & Ivanchenko, N. (2023). Trace metals in surface water of the Pechora River and its tributaries: Content, water quality and risks assessment (Arctic Ocean basin). *Marine Pollution Bulletin,**194*(6), 115317. 10.1016/j.marpolbul.2023.115317 37487428 10.1016/j.marpolbul.2023.115317

[CR35] Zaynab, M., Al-Yahyai, R., Ameen, A., Sharif, Y., Ali, L., Fatima, M., et al. (2022). Health and environmental effects of heavy metals. *Journal of King Saud University-Science,**34*(1), 101653. 10.1016/j.jksus.2021.101653

[CR36] Zeng, L., Yan, C., Yang, F., Zhen, Z., Yang, J., Chen, J., et al. (2023). The effects and mechanisms of pH and dissolved oxygen conditions on the release of arsenic at the sediment–water interface in Taihu Lake. *Toxics*. 10.3390/toxics11110890 10.3390/toxics11110890 PMC10675530 37999542

